# Appendiceal Neoplasm: It's Never What It Seems

**DOI:** 10.7759/cureus.65329

**Published:** 2024-07-25

**Authors:** Rita Marques, Urania Fernandes, Ricardo Vaz Pereira, Cátia Ferreira, João Pinto-de-Sousa

**Affiliations:** 1 General Surgery, Centro Hospitalar de Trás-os-Montes e Alto Douro, Vila Real, PRT; 2 Surgery, Clinical Academic Centre Trás-os-Montes e Alto Douro, Vila Real, PRT

**Keywords:** brief review, signet ring cells, neoplasm, mucinous, appendiceal

## Abstract

Appendiceal neoplasms are a rare and heterogeneous condition with a non-specific clinical presentation, often concealing intricate predicaments; hence they require complex and diverse management. The present case report aims not only to describe a less usual form of presentation and its orientation but also to provide a modest literature review in a rather demanding pathology.

## Introduction

Appendiceal neoplasms are rare (0.5-1% of intestinal neoplasms) [1-2] mainly divided into neuroendocrine (65%) and adenocarcinomas (20%) and within these mucinous. These neoplasms are reported to have a higher incidence in the white population but are not as expressive in women [2]. Clinical presentation can be vague and unspecific with subsequent delayed diagnosis or can be mistaken for acute appendicitis, especially in early stages (32%) [2]. Incidental diagnosis (imaging, surgical, or histological) is not negligible [2,3].

Treatment is highly dependent on the diagnostic timing, varying from appendectomy to more extensive organ resection and prognosis is greatly dependent on the disease stage. The presence of signet ring cells implies a poor prognosis (20% 5-year survival) [1].

## Case presentation

Herein the case of a 50-year-old female patient is presented. She reported a previous cesarean section but no other medical history. Family history highlights her father with colon cancer diagnosed at 80 years old and her brother with rectal cancer diagnosed at 48 years old (genetic test negative).

In the course of routine lower endoscopy, content nominated as purulent was observed in the appendiceal orifice without other lesions, and therefore, the patient was referred to the emergency department. There was no history of abdominal pain, nausea, vomiting, anorexia, fever, bowel habit alteration, or blood loss.

On physical exam, she presented with hemodynamic stability and had no fever and abdomen pain, palpable mass, or peritoneal signs. Blood work revealed no leukocytosis and normal C-reactive protein (Table 1).

**Table 1:** Blood work results

Blood work	
White blood count	9.590 x10^3^/uL (4.0-11.0 x10^3^/uL)
C-reactive protein	0.50 mg/dL (< 0.5mg/dL)

**Figure 1:**
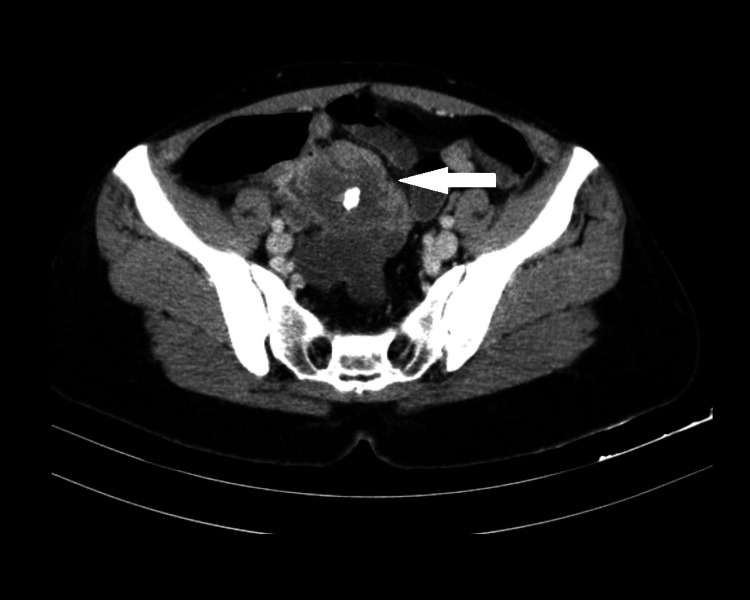
Abdominal-pelvic CT scan

An abdominal-pelvic computed tomography (CT) scan was performed which revealed a calcified nodular structure in the pelvic cavity with no cecum and right ovarium cleavage, measuring 82x60mm. A minimal amount of fluid in the pouch of Douglas and multiple enlarged adjacent lymph nodes were also identified (Figure 1).

Given the absence of other signs of appendicitis, the decision was to delay surgery in order to complete the investigation.

**Figure 2:**
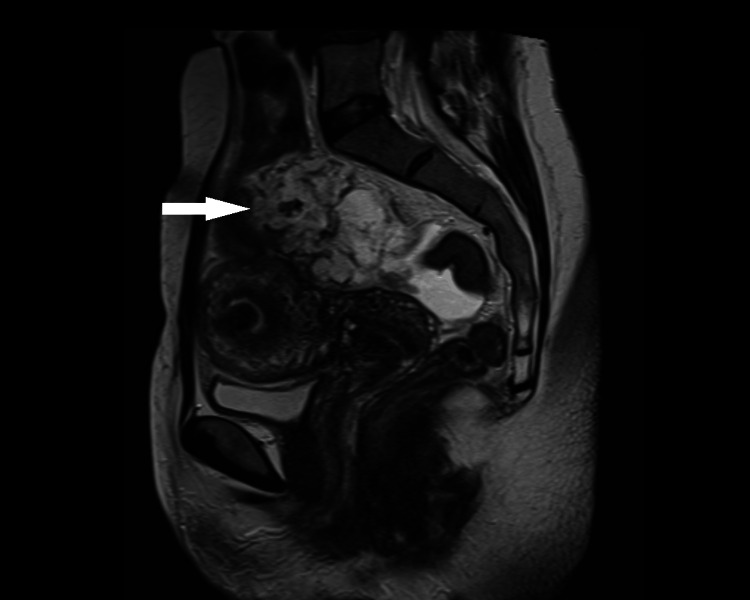
Abdominal-pelvic MRI

In an outpatient clinic, a chest CT scan was performed without evidence of disease. An abdominal and pelvic magnetic resonance imaging (MRI) identified an atypical septate complex cystic lesion, adjacent to the bladder dome and uterus, measuring 80x60mm, without direct invasion of the contiguous small bowel and signs of peritoneal disease (Figure 2).

Elevation of tumoral markers, namely carcinoembryonic antigen (CEA) and CA 125, was identified, with normal CA 19.9 (Table 2).

**Table 2:** Tumoral markers

Tumoral markers	
Carcinoembryonic antigen (CEA)	27.9 ng/mL (< 3.0 ng/mL)
CA 19.9	2 U/mL (< 37 U/mL)
CA 125	92 U/mL (< 35 U/mL)

Gynecological evaluation showed no alterations in the left ovarium with no identification of the right ovarium. After discussing with the institutional multidisciplinary oncology board, and due to doubt about the precise tumor origin, it was decided to perform laparotomy with resection of the involved organs if feasible.

Intra-operatively, no peritoneal or hepatic metastasis was identified, and the tumor involved the ascending colon (appendix not identifiable), right ovarium, uterus, and sigmoid colon.

**Figure 3:**
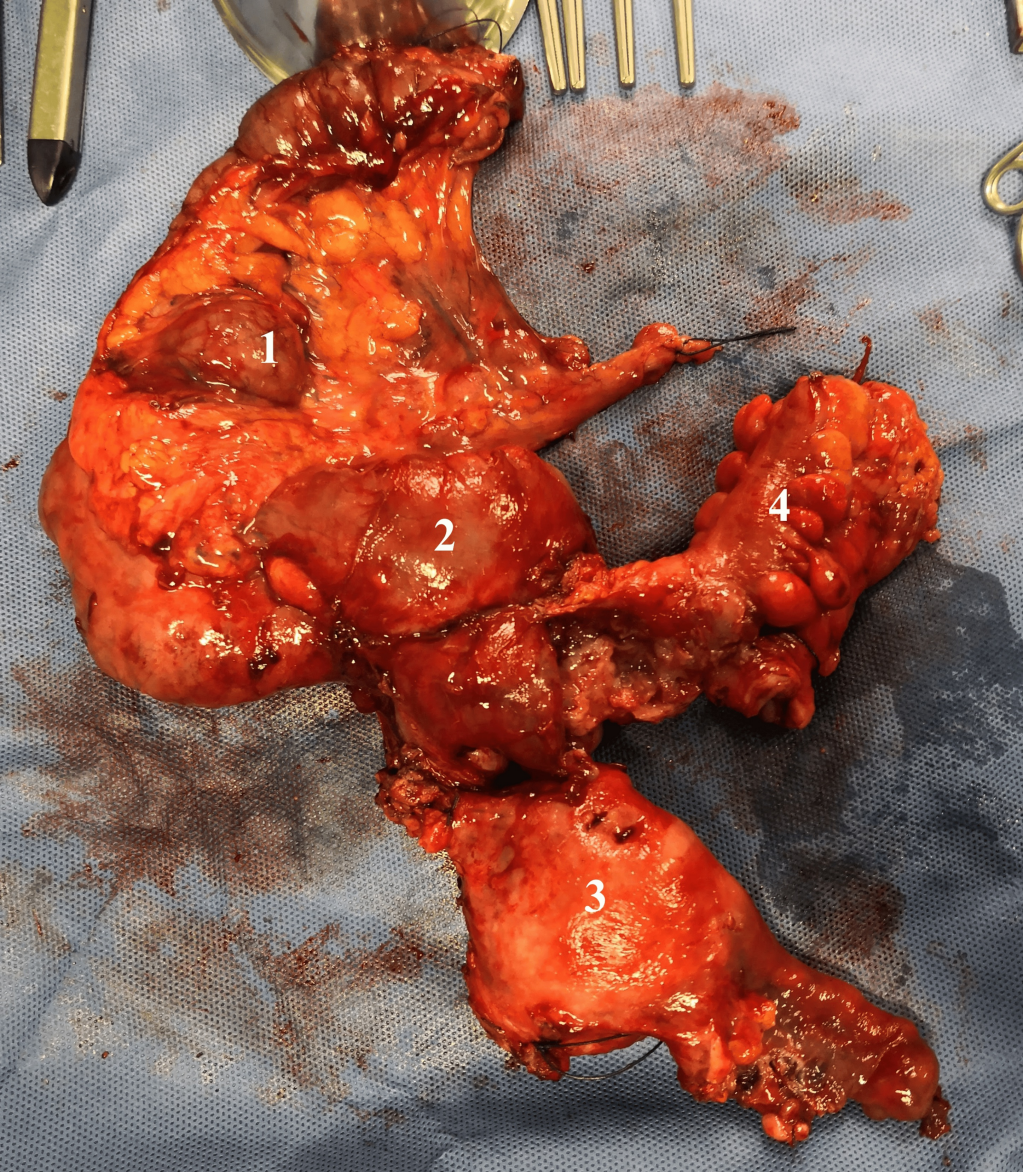
Surgical specimen 1: Ascending colon; 2: fused appendix and right ovarium; 3: uterus; 4: sigmoid colon

A multiorgan resection (right hemicolectomy, total hysterectomy with bilateral oophorectomy, sigmoidectomy, and omentectomy) was performed with an uneventful post-operative period (Figure 3).

**Figure 4:**
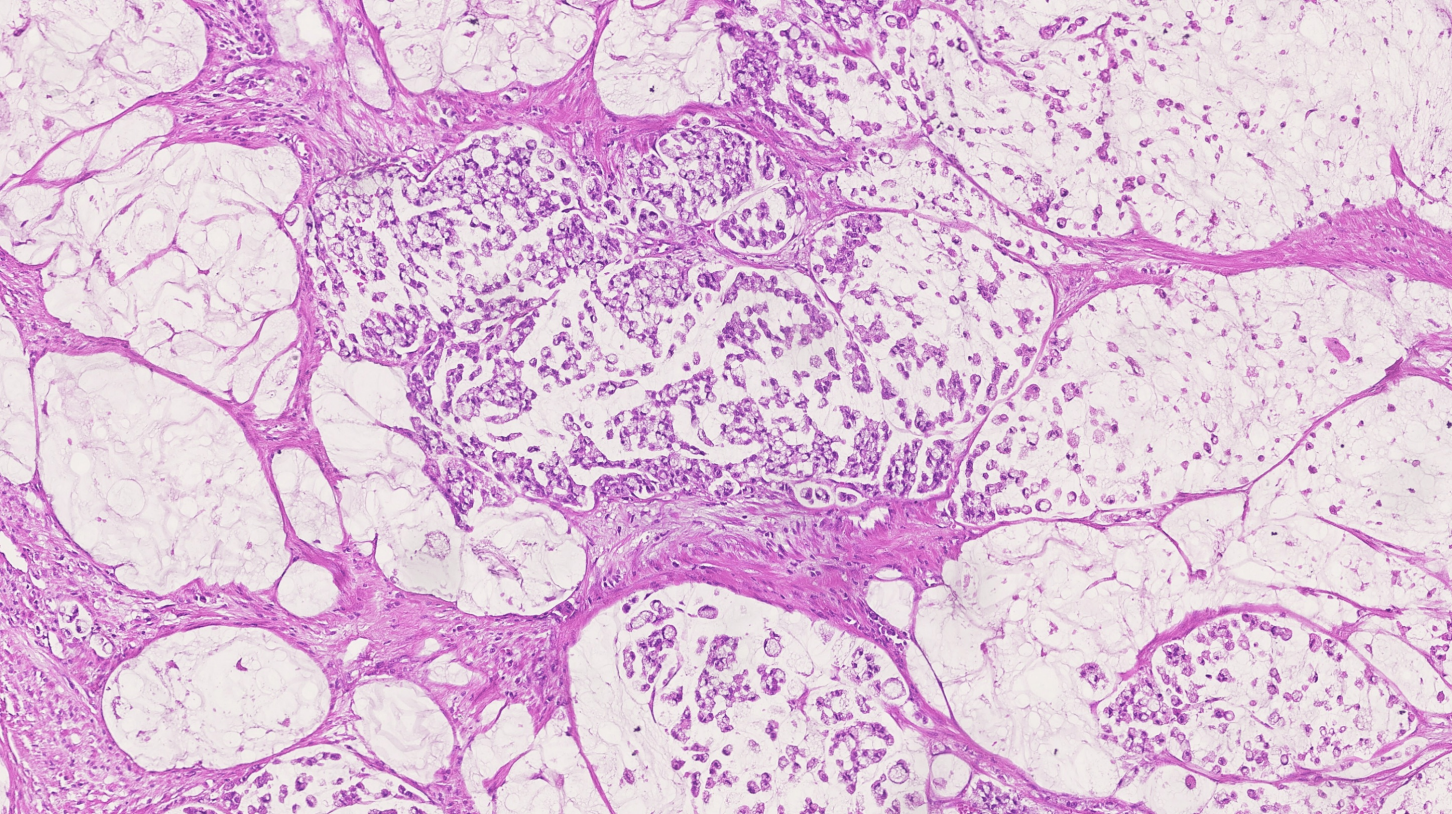
Appendiceal mucinous adenocarcinoma poorly differentiated with signet ring cells (hematoxylin and eosin stain)

The patient was discharged on day 6. Histology confirmed a poorly differentiated appendiceal mucinous adenocarcinoma with signet ring cell, staged as pT3 N0 (0/12) with adherence to the uterus, right ovarium, and sigmoid colon but without direct invasion and obvious perforation or extraluminal mucin (Figure 4). No lymphatic, venous, or perineural invasion was observed.

The case was re-discussed in the multidisciplinary oncology board with the decision to perform adjuvant chemotherapy (XELOX four cycles followed by four cycles of capecitabine). The 24-month follow-up showed no disease progression.

## Discussion

The present case recants the typical surgeon’s dilemma: the dichotomy between an acute case (appendicitis) and a lengthy and complex situation (a neoplasm) requiring a more cautious approach.

This differentiation can be amenable pre-operatively (through endoscopy and imaging), intra-operatively, or only post-operatively (through histopathological evaluation). Nonetheless, a big percentage is still mistakenly masked as acute appendicitis [1] or incidentally during surgery for other purposes, posing orientation challenges. More advanced stages usually present with constitutional symptoms and signs of ascites [2].

In this case, the absence of other signs of appendicitis (anorexia, nausea, vomiting, abdominal pain, or fever) combined with no acute inflammatory biomarkers as well as no CT scan signs of appendicitis precluded an urgent approach.

The investigation, targeted to the likelihood of neoplasm, included a CT scan but also an MRI given its superiority in detecting extraluminal mucin and peritoneal disease [3-7].

Hence, staging confirmed the possibility of a tumor arising from the appendix or right ovarium and no biopsy was considered due to the risk of neoplastic seeding. Therefore, surgery was necessary to enlighten the diagnosis [3-7].

Intra-operatively the absence of signs of peritoneal disease allowed for a wide dissection. The decision to perform extensive gynecological surgery was based on the non-identification of the right ovarium (likelihood of 48.6% of microscopical involvement) [7,8] as well as a lack of clear etiology. Furthermore, sigmoidectomy was necessary due to suspicion of direct invasion.

The histology report confirmed the appendiceal origin, namely a poorly differentiated mucinous adenocarcinoma with signet ring cells, which implies > 50% of extracellular mucin but ≤ 50% of signet ring cells [3-6].

This pathology comprises a higher probability of spread and consequently a worse prognosis (20-25% 5-year survival) [1,6,7], a condition not verified in our patient with locally advanced tumor without spread.

The indication for cytoreductive surgery (CRS) and hyperthermic intraperitoneal chemotherapy (HIPEC), recommended in fit patients with resectable peritoneal disease, may be disregarded in the face of aggressive histology as signet ring cells, even though it is not considered futile [7]. This case had no indication for CRS and HIPEC due to the absence of peritoneal disease, as previously described.

The role of adjuvant chemotherapy in appendiceal tumors is extrapolated from the results of colon adenocarcinoma, with fluorouracil and oxaliplatin being considered valid options [1]. Nonetheless, this evidence is directed to adjuvant treatment after CRS and HIPEC but has a particular benefit in aggressive histology as signet ring cells [7]. Regardless, an indication of adjuvant treatment in colon cancer is particularly dependent on pathological nodal status, which doesn´t appear to be transposable to this pathology [9].

In this case, given the absence of peritoneal disease, the decision was made to perform adjuvant chemotherapy with capecitabine and oxaliplatin with good results to date, proven by the 24-month innocent follow-up.

This case has many remarkable aspects such as the patient being asymptomatic [10] and presenting with multiorgan involvement even though without peritoneal spread (53.2% present with extensive peritoneal disease at diagnosis) [2-10].

Continuity of follow-up (both by an oncologist and a Colorectal surgeon) is paramount, given the possibility of recurrent disease (25% after CRS and HIPEC) [7] especially intraperitoneal. However, there is no standardized time length, although a minimum of 6 years in high-grade tumors is generally accepted, such as in this case [7]. In the event of intraperitoneal recurrence, CRS and HIPEC should be re-equated [10].

## Conclusions

Appendiceal neoplasms should be part of the surgeon’s diagnostic arsenal, given the prospect of changes in intra-operative decisions that largely affect patient survival. Their histological diversity poses a true challenge in treatment, and mucinous adenocarcinomas, specifically with signet ring cells, emphasize this concern, mainly due to their mediocre prognosis.

Further prospective trials are mandatory to delineate more concrete guidelines for surgical patients (namely the indication for neoadjuvant therapy) as well to better clarify the role of chemotherapy for non-surgical patients (particularly palliative therapy) as well to standardize follow-up.
